# Lunacy in Scotland

**Published:** 1892-07-23

**Authors:** 


### Lunacy in Scotland.

The last published annual report, being the thirty-third, of the Commissioners in Lunacy for Scotland, is, as usual, a most interesting and ably prepared volume. It is divisible into three sections, the first containing the report proper, and is addressed to the Marquis of Lothian in his capacity of Secretary of State for Scotland. The second part runs to forty-two pages, and is entirely composed of statistical tables, while the third consists of entries left by the Commissioners at the various institutions visited by them.

On the date of publication of this blue book there were in Scotland 10,593 registered lunatics. Of these, 5,589 were in public asylums; 2,399 were in workhouses; 2,489 were in private dwellings, and only 152 were in private asylums. Contrasting this state of things with what pertains in England, we see that upwards of 54,000 of the total of 86,000 were in county or borough asylums; 4,500 were in private asylums; 3,688 were in lunatic hospitals; 6,200 were in private dwellings; and about 17,000 either in workhouses or in kindred institutions. In Scotland there was not a single pauper lunatic in a private asylum, whereas there were in England 1,690 paupers, supported at great additional expense by the ratepayers, in English private asylums. We have ere now commented on the wrong suffered by the tax-payer through this English custom, and we draw the attention of our readers once more to it in the hope that the question may be ultimately taken up by our legislators, and an end put to a proceeding which is as unnecessary as it is expensive.

During the year there was an increase of 282 registered lunatics, an increase which must be looked on as large for Scotland, and for which there does not seem to be any recognisable reason.

One of the best points of Scotch lunacy administration is the permission given to patients to reside in the asylums as voluntary boarders. The Commissioners say that no difficulty or disadvantage has resulted from the association of the registered insane with these voluntary boarders; and when we learn that 98 persons availed themselves of the privilege we can easily understand how much it is appreciated. These people obtained all the benefits of asylum treatment "in a way which is not attended with troublesome or disagreeable forms."

There were 110 accidents, nine being fatal and including three suicides, the latter being relatively three times as numerous as during the same period in English asylums. This fact may mean that the English suicidal lunatics are more carefully supervised than the Scotch, or that the Scotch run the risk of extra suicides for the enormous advantages which additional freedom confers on the melancholic cases generally; or it may be that the numbers are exceptionally high on one side and exceptionally low on the other, or possibly the Scotch suicidal case has more determination than his English brother. The statistics-monger can choose for himself.

The recoveries range from 35 per cent. of the admissions in private asylums to 46 per cent. in parochial asylums; the Royal and district asylums steering a middle course with 38 per cent. These differences are chiefly, if not entirely, owing to the different nature of cases admitted and not to any superiority of treatment practised in the asylums.

The death-rate is lower than the average in England, being 8·4 for private patients and 8·1 for paupers; the English average being about 10 per cent. Of the total number of deaths in Royal and district asylums 13·4 per cent. of the males and 18·7 per cent. of the females were caused by consumption.

It will be seen from the second paragraph of this notice that although the gross number of lunatics in Scotland is only one-eighth of the English total, the number of lunatics boarded in private dwellings is nearly 2,500, while the number of patients so treated in England only reaches 6,200. The Scotch Commissioners have always encouraged this system, and the result is that upwards of one-fourth of the insane are kept out of the asylums, and the asylum themselves are kept within reasonable limits. In England we have only about one-fourteenth of our total number in private dwellings, and could the Scotch system be got to take proper root in England, something like fifteen thousand lunatics might be drafted from our congested mammoth asylums. And if at the same time the Irish and Scotch patients could be sent to their native climates, an end would be put, for many a year to come at least, to that seemingly everlasting mess of bricks and mortar which surrounds our county and borough asylums.

When speaking of the Royal Asylums, the Commissioners say they "think it necessary to repeat the statement made in last year's report that they cannot be regarded as having done all that ought to be done, nor all that can be done if public attention is intelligently directed on the matter, until all patients for whom rates of board of not more than £25 a year can be paid are provided for in these institutions as private patients." The Royal asylums somewhat resemble our lunatic hospitals, and it is greatly to be hoped they will not follow in the wake of so many of ours, and admit the richer lunatic to the exclusion of the comparatively poor; but the Commissioners' stricture would seem to apply rather to the public for not subscribing than to the managers for not admitting as many as they can.

Scotland is now expending a total of £236,000 a year on the maintenance of her pauper lunatics, being about 9s. per head per week. In England the cost per head per week is about 8s. 7d. in county asylums and 10s. in borough asylums.

